# Tuberculosis and Indoor Biomass and Kerosene Use in Nepal: A Case–Control Study

**DOI:** 10.1289/ehp.0901032

**Published:** 2009-12-17

**Authors:** Amod K. Pokhrel, Michael N. Bates, Sharat C. Verma, Hari S. Joshi, Chandrashekhar T. Sreeramareddy, Kirk R. Smith

**Affiliations:** 1 School of Public Health, University of California–Berkeley, Berkeley, California, USA; 2 Regional Tuberculosis Center, Ram Ghat, Pokhara, Nepal; 3 Department of Community Medicine, Manipal Teaching Hospital, Manipal College of Medical Sciences, Pokhara, Nepal

**Keywords:** biomass fuel, cooking-fuel smoke, heating, indoor air pollution, kerosene lighting, kerosene stove, smoking, women

## Abstract

**Background:**

In Nepal, tuberculosis (TB) is a major problem. Worldwide, six previous epidemiologic studies have investigated whether indoor cooking with biomass fuel such as wood or agricultural wastes is associated with TB with inconsistent results.

**Objectives:**

Using detailed information on potential confounders, we investigated the associations between TB and the use of biomass and kerosene fuels.

**Methods:**

A hospital-based case–control study was conducted in Pokhara, Nepal. Cases (*n* = 125) were women, 20–65 years old, with a confirmed diagnosis of TB. Age-matched controls (*n* = 250) were female patients without TB. Detailed exposure histories were collected with a standardized questionnaire.

**Results:**

Compared with using a clean-burning fuel stove (liquefied petroleum gas, biogas), the adjusted odds ratio (OR) for using a biomass-fuel stove was 1.21 [95% confidence interval (CI), 0.48–3.05], whereas use of a kerosene-fuel stove had an OR of 3.36 (95% CI, 1.01–11.22). The OR for use of biomass fuel for heating was 3.45 (95% CI, 1.44–8.27) and for use of kerosene lamps for lighting was 9.43 (95% CI, 1.45–61.32).

**Conclusions:**

This study provides evidence that the use of indoor biomass fuel, particularly as a source of heating, is associated with TB in women. It also provides the first evidence that using kerosene stoves and wick lamps is associated with TB. These associations require confirmation in other studies. If using kerosene lamps is a risk factor for TB, it would provide strong justification for promoting clean lighting sources, such as solar lamps.

Tuberculosis (TB) is a major infectious disease that causes illness and death worldwide (Rieder 1999). In 2006, there were about 9.2 million new TB cases and 1.7 million TB-related deaths [World Health Organization (WHO) 2008]. Most new cases and deaths occurred in Asia and Africa. In Nepal, a South Asian country, TB is a major public health problem (Paugam and Paugam 1996), with an overall annual incidence of all forms of TB estimated at 176 per 100,000 persons (Harper et al. 1996).

A range of social, environmental, and behavioral factors influence exposure and susceptibility to *Mycobacterium tuberculosis* infection. Identifying TB risk factors and minimizing exposure to them could reduce the TB burden in Nepal and other developing countries. Active tobacco smoking, for example, has been shown to be a risk factor for TB, presumably by damaging immune and other protective mechanisms, allowing TB infection to prosper (Bates et al. 2007; Boelaert et al. 2003; Lin et al. 2007). The composition of tobacco smoke has many similarities to that of indoor cooking smoke from biomass fuel (Kulshreshtha et al. 2008; Shalini et al. 1994; Smith 1987), exposure to which is common in the developing world, including Nepal. Therefore, an association of TB with indoor cooking smoke is plausible. Six previous epidemiologic studies have investigated whether an association exists between TB and exposure to cooking-fuel smoke (Crampin et al. 2004; Gupta et al. 1997; Kolappan and Subramani 2009; Mishra et al. 1999; Padilla et al. 2001; Shetty et al. 2006). Although four of these studies found some evidence of an association, all the studies had limitations. The first study to find an association between exposure to cooking-fuel smoke and TB presented limited data on potential confounding factors, and the risk model was adjusted only for age, which left open the possibility of confounding by socioeconomic factors or smoking (Gupta et al. 1997). Mishra et al. (1999) also reported evidence of an association; however, they used data from the 1992–1993 Indian National Family Survey, which was based on self-reported TB status. This leaves the possibility of outcome misclassification. A third study found an association between cooking smoke exposure and TB but included no validation of key components of the questionnaire (Padilla et al. 2001). In a study conducted in Malawi, Crampin et al. (2004) found no association between cooking smoke exposure and TB, but the study participants varied little in the type of fuel they used, and the risk model was adjusted only for age, sex, area of residence, and HIV status, leaving open the possibility of confounding by other socioeconomic factors or smoking. The fifth study, conducted in South India by Shetty et al. (2006), also found no association of cooking-fuel smoke with TB, but they did find an association between TB and not having a separate kitchen. The sixth study was conducted by Kolappan and Subramani (2009) in Chennai, India; they found a marginal association between biomass fuel and pulmonary TB in their study population [adjusted OR = 1.7; 95% confidence interval (CI), 1.0–2.9]. The study participants in this study were primarily men (87%) but because women do most of the cooking, they are more likely to be exposed to smoke from cooking fuel.

We conducted a TB case–control study in the Pokhara municipality of Nepal where cooking with biomass fuels in unvented indoor stoves is a common practice. Our main objectives were to confirm results of earlier studies using clinically confirmed TB cases and to investigate possible confounding of the relationship using a validated questionnaire and exposure assessment in the kitchens of a subset of participants’ houses.

## Methods

Subjects’ approvals were obtained from the institutional review boards at the University of California–Berkeley, and at the Nepal Health Research Council.

The study was conducted at the Regional Tuberculosis Center (RTC) and the Manipal Teaching Hospital (MTH), Manipal College of Medical Sciences, in Pokhara. The RTC and MTH are the two major health centers [directly observed treatment short-course (DOTS) clinics] that specialize in diagnosing TB and caring for people who live in Kaski (Pokhara) and seven adjoining hill districts: Syangja, Parbat, Tanahu, Lamjung, Myagdi, Baglung, and Gorkha, which are in the midwestern development region of Nepal. All subjects were recruited and interviewed between July 2005 and April 2007. The climate of the region is temperate but can be cool at times. For example, in Pokhara city (latitude 28.2° N), which is 827 m above sea level (Central Bureau of Statistics 2009), the mean temperature and mean daily minimum temperatures in January 2006 (the coldest month of the year) were 14.3°C and 7.2°C, respectively (Department of Hydrology and Meteorology 2006/2007). Other, more elevated parts of the region can be colder.

### Recruitment procedure for cases and controls

Cases were all female patients, 20–65 years old, who visited TB clinics in RTC (90.4%) and MTH (9.6%) and who had been newly diagnosed with active pulmonary TB by chest X-ray and positive active sputum smears (two sputum specimens positive for acid-fast bacilli by microscopy), which are routinely conducted at the hospital using methods recommended by the WHO (1997). Women who were pregnant, who were on chemotherapy for cancer, who had HIV/AIDS or diabetes, or who had a history of TB were excluded from the study.

Controls were recruited from outpatient and inpatient departments (dental, 1.6%; ear, nose, and throat, 1.6%; ophthalmology, 25.6%; general medicine, 56%; obstetrics and gynecology, 7.2%; orthopedics, 2.4%; skin, 1.6%; surgery, 3.2%; and psychiatry, 0.8%) at the MTH, in the same months when cases were identified. For each case, the control subjects were the first eligible female patients without pulmonary TB, matched to cases on age (5-year frequency bands), who presented at MTH between 0900 and 1000 hours after case enrollment. Controls were excluded from the study for the same reasons as for the cases. Control subjects were interviewed only after medical screening confirmed that they did not have TB. Confirmation procedures included a chest X-ray and an on-the-spot sputum examination. The ratio of cases to controls was 1:2.

After obtaining an informed oral consent to participate, all cases and controls were interviewed face-to-face by trained interviewers shortly after diagnosis while they were still at the hospital. The three interviewers were unavoidably aware of the case or control status of the interviewees but were not aware of the main exposure of interest or hypothesis of the study. All interviewers interviewed both cases and controls.

The questionnaire collected data on education level, area of residence (urban, periurban, and rural), history of use of cooking fuels and stoves that included present and previous (including in parents’ houses, before marriage) cooking fuels and stoves, present kitchen type and location, kitchen ventilation, house type, participant’s smoking history and smoking status of family members, alcohol consumption, vitamin supplement consumption, use of mosquito coils and incense, household crowding, vehicle ownership, and annual income level.

### Statistical analysis

Liquefied petroleum gas (LPG) and biogas were designated “gaseous-fuel stoves” (GFS), which was used as the reference category for most analyses compared with kerosene-fuel stoves (KFS) and biomass-fuel stoves (BFS). Very few participants (two cases and four controls) reported burning biomass in stoves with flues or chimneys venting to the outside, and no one reported using an electric cooker. For this reason, no separate category was created for vented BFS, and these subjects were included in the BFS category.

We examined the extent of agreement of responses on the exposure information (current stove/fuel type and ventilation) obtained during face-to-face interviews at the hospital with data obtained from actual inspection of these features in the houses of the first 28 study participants (13 cases and 15 controls). The effect of misclassification was calculated in terms of sensitivity and specificity.

We combined information on kitchen location and windows in the kitchen to create a composite dichotomous variable for ventilation. “Fully and partially ventilated kitchens” included open-air kitchens, separate kitchens outside the house, and partitioned kitchens with windows inside the house. This was used as the reference category for ventilation. Unventilated kitchens included partitioned and nonpartitioned kitchens without windows inside the house. We were unable to clearly interpret questionnaire data on closing doors in a way that could be used to characterize ventilation.

To calculate the number of pack-years of smoking, we combined the information on the average number of tobacco products (cigarettes or *bidis*) smoked every day multiplied by the duration of smoking in years divided by 20, assuming that a pack of cigarette contains twenty cigarettes/*bidis*. One participant who reported she smoked a *hukka* (water pipe) was excluded from this analysis.

We calculated crude odds ratios (ORs) between exposure and outcome. We decided a priori to include all statistically significant (*p* ≤ 0.05) variables in the model, as well as any other recognized risk factors for TB. Then we applied a stepwise backward elimination model, with a variable selection criterion of *p* = 0.2, to all the variables to identify any others that should be included in the final model. Using the selected covariates, we constructed a multivariate unconditional logistic regression model for risk of TB. We calculated adjusted female population-attributable fractions and associated CIs using the aflogit command in Stata (version 10; StataCorp LLC, College Station, TX, USA) statistical software (Eide 2008). This procedure assumes that the proportion of controls exposed is a good estimate of the proportion exposed in the target population.

## Results

Four potential interviewees (all cases) did not meet the inclusion criteria: two were diabetic and two were HIV positive. During recruitment, one potential control was found to have pulmonary TB and was transferred to the case group. Except for one control, all potential interviewees agreed to participate in this study. In total, we recruited and interviewed 125 cases and 250 controls. Cases were more likely to be referred by a health care professional (30.4%) than were controls (7.2%). This might reasonably be expected because TB causes serious illness, but many of the controls would have had much less severe conditions.

Table 1 lists descriptive data for the cases and controls, with unadjusted ORs and CIs. With the exception of the income variable, few data were missing. Confirming the success of the matching process, distributions of cases and controls were similar in terms of age. Most cases and controls (72.0% of cases, 94.4% of controls) were from the Kaski district. Cases were more likely than controls to be Buddhist, to live in urban and periurban areas, to reside in poorer quality houses (*kuccha*), to be illiterate, to have nonpartitioned and unventilated kitchens indoors, and to use kerosene wick lamps as their main source of light. Cases were also more likely than controls to regularly consume alcohol, to be tobacco smokers, to have more smokers in the family than controls, and to have not always lived in their present house. We think that, to some extent, the latter variable probably captures the likelihood of previously having used other cooking fuels. Except for three cases, none of the participants who had smoked reported that they had ever quit smoking for 6 months or more. Therefore, we classified smokers as ever-smokers and never-smokers. The median smoking experience for both cases and controls was 8 pack-years (SD = 13.37 pack-years). More cases than controls had had household members with TB. Moreover, cases were more likely to be using BFS or KFS than were controls (*p* = 0.004). The distribution of cooking fuel used by the study participants was biomass from wood or crop residues (44.3%), LPG (42.7%), kerosene (11.2%), and biogas (1.9%).

We created a heating fuel variable that treated participants who reported either using electricity (1 case, 3 controls) or using no heating fuel (38 cases, 137 controls) as the reference category, and the remaining subjects, who mainly used wood (84 cases, 107 controls), as the biomass fuel category. The biomass group included a few women who used coal (one control) and kerosene (one case, one control) for heating.

We verified stove-fuel types and ventilation characteristics in the houses of 28 participants. All 18 participants who had reported their main cookstove as being a biomass stove were found to be correct, as were the five reporting use of a LPG stove. One of the four participants who had reported using a kerosene stove, however, was found to be using an LPG stove. On that basis, the accuracy (true reports ÷ total reports) of stove reporting was 96%. In the inspection of ventilation characteristics, one participant who had reported not having a window in her kitchen was found to have a temporary outside kitchen with a window-sized opening. Two participants who reported having a window in the kitchen actually did not have a window. Based on these data, the accuracy for reporting ventilation was 89%.

As shown in Table 1, the unadjusted exposure ORs for cooking in BFS and KFS were 1.98 (95% CI, 1.24–3.17) and 2.54 (95% CI, 1.26–5.12), respectively. Use of kerosene lamps had an unadjusted OR of 10.35 (95% CI, 3.42–31.3), and use of biomass fuel for heating had an OR of 2.81 (95% CI, 1.78–4.42). Compared with cooking in a fully ventilated or partially ventilated kitchen, cooking in an unventilated kitchen was associated with a doubling of the risk of TB (OR = 2.02; 95% CI, 1.31–3.13).

The univariate analysis showed statistically significant (*p* ≤ 0.05) associations of TB with use of mainly biomass, coal, and kerosene as a source of heating fuel, urban/rural locality of residence, residence outside the Kaski district, religion, literacy, construction type of present house, not always having lived in the present house, ventilation, use of a kerosene lamp, tobacco smoking, one or more smokers in the family, alcohol consumption, vitamin consumption, and having had a family member with TB. Although not selected by the stepwise algorithm, in the multivariate model we also included annual family income in Nepali rupees as an additional indicator of socioeconomic status, and age, because it was a matching variable. Table 2 shows the results of the main logistic regression model. Compared with use of GFS, use of a biomass-fueled stove for cooking showed a slight positive relationship, but the CI was so wide that this provides little evidence of an association with TB. Kerosene cooking-fuel use, however, was associated with TB. Also particularly strongly associated with TB in the model were use of biomass as a heating fuel (OR = 3.45; 95% CI, 1.44–8.27) and kerosene lamps as the main source of lighting in the house (OR = 9.43; 95% CI, 1.45–61.3).

We investigated possible effect modification of the biomass fuel variables by other exposures. However, investigation was limited because of small numbers of participants in many of the exposure categories, leading to very unstable estimates. Covariates with sufficient numbers in separate categories permitting some useful examination of effect modification were ventilation, literacy, and house construction. We found evidence of effect modification of the effects of heating fuel by ventilation status: participants who lived in houses with unventilated kitchens were at much higher risk (adjusted OR = 26.0; 95% CI, 4.24–159) than were those who lived in houses with ventilated kitchens (adjusted OR = 7.07; 95% CI, 1.48–33.9). Corresponding estimates for biomass cooking fuel were much more equivocal, with the adjusted ORs for ventilated and unventilated kitchens being 0.80 (95% CI, 0.19–3.37) and 0.47 (95% CI, 0.08–2.94), respectively. For illiterate and literate participants, adjusted ORs for heating fuel use were 5.12 (95% CI, 0.96–27.4) and 2.93 (95% CI, 0.87–9.91), respectively. We found no evidence of effect modification of literacy status on biomass cooking-fuel effects. Finally, participants who lived in *kuccha* construction houses (bamboo and mud, with thatched roofs) appeared to be at higher risk from both biomass cooking and heating fuels than were participants who lived in *pucca* or semi-*pucca* construction houses (brick and cement or brick and mud). For heating fuel, the adjusted ORs were 11.9 (1.38–102) and 2.73 (0.88–8.41) for *kuccha* and *pucca*/semi-*pucca* houses, respectively. The corresponding values for biomass cooking-fuel use were 4.07 (95% CI, 0.43–38.8) and 0.73 (0.22–2.40), respectively. With the possible exception of the modification by ventilation of the effects of biomass cooking fuel, these effects might generally be considered to be in the predictable direction—higher ORs associated with less ventilation and more-deprived socioeconomic circumstances.

### Exposure response

We investigated whether associations with TB varied according to duration of cooking with BFS or KFS (Table 3). We categorized the total durations of cooking on BFS and KFS by cases and controls into bands. The adjusted exposure ORs were 1.17 (95% CI, 0.32–4.32), 0.64 (95% CI, 0.18–2.20), and 0.47 (95% CI, 0.11–2.02) for use of a BFS for less than 5 years, 5–10 years, and >10 years, respectively. For KFS, the unadjusted ORs were 4.96 (95% CI, 1.44–17.1) and 4.60 (95% CI, 1.34–15.7) for less than and more than 5 years of use, respectively, relative to no KFS use. Because we did not collect duration data for either heating fuel use or household lighting, we could not carry out comparable analyses for these variables.

As one measure of the potential public health implication of the association, we estimate that the population-attributable fractions of TB from exposure to BFS, KFS, biomass fuel heating, and kerosene lamps in our target population were 9% (95% CI, –42% to 41%), 12% (0.2–22%), 47% (22–64%), and 13% (4–22%), respectively.

## Discussion

The results of this study suggest that indoor exposure to smoke from biomass fuel combustion is a risk factor for TB. The association, however, appears to be mainly with use of biomass for heating, rather than cooking. The study also strongly suggests that exposure to smoke from kerosene fuel combustion, either in stoves or in lamps, is a risk factor for TB.

Religion, income, residence outside Kaski district, vitamin consumption, a family history of TB, and not always having lived in the present house also showed statistically significant associations with TB (Table 1). Pack-years of smoking (> 8 pack-years) showed an association with TB (*p* = 0.06), which did not change appreciably after adjustment. Smoking is now an established risk factor for TB (Bates et al. 2007; Chiang et al. 2007; Leung et al. 2004; Slama et al. 2007; Yu et al. 1988). The very elevated relative risk estimate for Buddhists relative to Hindus is striking. We considered the possibility that this may have been because some Buddhists who live around Pokhara are Tibetan and reside in refugee camps. Crowded conditions in those camps could facilitate TB transmission. However, only 8 of 40 Buddhists in the study (six cases, two controls) were Tibetan refugees—an insufficient number to explain the finding. Other studies have also shown differences in TB rates between racial and religious groups, including Tibetan Buddhists (Bhatia et al. 2002; Hill et al. 2006; Mishra et al. 1999; Nelson et al. 2005; Truong et al. 1997).

Before concluding that statistical associations are causal, it is important to consider alternative explanations, particularly whether study results might be a result of selection bias, information bias, or confounding in the study design, data collection, or analysis. As with all case–control studies, selection bias in the recruitment of controls is a potential concern. In this study, a systematic procedure for recruitment of all controls from inpatient and outpatient departments of MTH was used, and only one potential control refused to participate. Because most cases were recruited from the RTC, and all controls from MTH, the catchment areas for MTH and RTC might have been different. RTC patients came from a broader area, because it is a referral center for the western development region of Nepal. A higher proportion of cases (28%) than controls (6%) were from five districts other than Kaski. The Kaski district includes Pokhara city, and in general, Kaski residents are more likely to live in urban areas and to be wealthier. This could simply mean that living outside of Kaski is associated with higher exposure to TB risk factors but, alternatively, could indicate some selection bias. We adjusted for area of residence (Kaski or other districts) in the final model, but this would not necessarily have eliminated such a bias.

Another possible source of selection bias arises because we did not exclude some other, non-TB respiratory disease cases from the control group. Unfortunately, control diagnoses were not collected at the time of the study and proved impossible to obtain in retrospect, because of the limited period for which the hospital retains patient records. Because absence of TB was confirmed in controls by X-rays, we can, however, be confident that no chronic obstructive pulmonary disease or pneumonia cases were among our controls. It is possible that inclusion of respiratory disease cases among the controls could have produced a bias toward the null, if risk factors for those cases were similar to risk factors for TB.

Information bias may take the form of outcome misclassification or exposure misclassification. Because all cases were newly diagnosed with active pulmonary TB on the basis of evidence from clinical tests, and controls were also confirmed by chest X-ray and on-the-spot sputum smear testing as not having active pulmonary TB, we consider that disease misclassification is unlikely to have occurred. We obtained all the exposure data by questionnaire. Case–control studies are often considered susceptible to recall bias, in that cases may be more likely than controls to remember past exposures. Because questions asked in this study were about common exposures, however, which both cases and controls experience on a day-to-day basis, we expect recall to have been accurate and any differential recall to have been minimal. We verified the high level of accuracy of reporting of two key exposure variables (stove type and ventilation) by visiting the homes of 28 study participants. Considering this, and that there is no prevailing belief that indoor smoke exposure from biomass-burning stoves or kerosene-burning stoves or lamps is related to TB occurrence, we believe exposure misclassification is likely to be minimal. One possible limitation, however, is that we only asked about the main cooking fuel used. This might have led to some misclassification of exposure status.

The third main area of potential bias is confounding. We collected data on a much more comprehensive range of exposures than did previous studies and investigated their potential to confound the associations with fuel use. Although confounding was present, adjustment with these variables did not eliminate the key associations. There may, of course, be some residual confounding due to misspecification of the variables, and there is no way to rule out the possibility of unknown confounding factors causing the associations found. One possibility is malnutrition, for which we obtained no data and which is a known risk factor for TB. However, family income, for which we did obtain data and which is an excellent indicator of a family’s ability to feed itself, was taken into account.

A notable finding in our study was the association with biomass used as a heating fuel. This was unexpected because the study design focused on cooking-fuel use. Hence, the study population was limited to women, who generally do the cooking in Nepal. Although we collected data on history of stove and cooking-fuel use, we did not collect a comparable level of data for heating fuels and so are unable to examine heating-fuel use for evidence of an exposure–response relationship.

In hindsight, the findings with biomass as a heating and a cooking fuel make sense. Women may light a cooking fire, set the pot atop it, and leave the room, returning only periodically while cooking takes place. On the other hand, use of heating fuel involves minimization of ventilation and deliberate exposure, as the family sits around the fire. In tropical India and Africa, where several of the other TB and biomass studies have been carried out, use of heating fuel is less common than in the mid-hills of Nepal, where nighttime and winter temperatures are lower.

Our study also found the OR for TB to be high among both kerosene stove and lamp users, particularly the latter. Kerosene cooking fuel and kerosene lamp users were for the most part mutually exclusive groups. Only one of the 22 kerosene lamp users in the study used a kerosene stove. Kerosene stove users were more likely to use electricity for lighting. With one exception, as far as we are aware, no previous studies have examined a relationship between kerosene and TB (Padilla et al. 2001). This one study, carried out in Mexico, obtained crude ORs for use of kerosene-burning stoves of 1.9 (95% CI, 0.8–4.5) for active TB and 4.4 (95% CI, 1.7–11.5) for past TB; no adjusted estimates were presented. We have been unable to find any studies where the relationship between kerosene lighting and TB has been investigated or even incidentally reported.

The question arises as to why kerosene as a cooking fuel could be a TB risk factor but not biomass cooking fuel. This could have something to do with the nature of the emissions. Biomass burning produces very obvious smoke, which may irritate the eyes and respiratory tract, encouraging avoidance behavior. Kerosene, on the other hand, has the appearance of burning more cleanly, even if it does produce substantial amounts of fine particulate matter and vapor-phase chemicals, and may not encourage the same avoidance behavior as biomass smoke. Cooks may be more likely to remain in the room while cooking with kerosene fuel. There are also likely to be differences in the toxic effects of the pollutant mixtures from the two fuels.

Kerosene is one of the main sources of cooking fuel in urban areas and lighting fuel in rural areas of developing countries, including Nepal. Therefore, if kerosene burning can be confirmed as a TB risk factor in other studies, the public health implications would be substantial. In rural areas not connected with electric power, kerosene wick lamps are burned at least 4–5 hr every day. Commonly, these lamps are homemade devices that are highly energy inefficient, with low luminosity. Simple wick kerosene lamps emit substantial amounts of smoke and particles (Schare and Smith 1995). A study conducted in rural Malawi has shown a higher loading of particulates in alveolar macrophages in men from exposure to kerosene in lamps compared with candles, hurricane lamps, and electric lamps (Fullerton et al. 2009). Other emissions from kerosene combustion include carbon monoxide, carbon dioxide, sulfur dioxide, nitrogen dioxide, formaldehyde, and various VOCs (volatile organic carbons) (Traynor et al. 1983). An indoor air pollution study conducted in Bangladesh slums has shown significantly higher concentrations of benzene, toluene, xylene, hexane, and total VOCs emitted from kerosene stoves than from wood-burning stoves (Khalequzzaman et al. 2007).

The use of kerosene fuel is associated with harmful effects that have been documented in a few studies. These effects include impairment of ventilatory function and a rise in blood carboxyhemoglobin in women exposed to kerosene fuel smoke (Behera et al. 1991), and a higher incidence of acute lower respiratory infection in children in homes using KFS and BFS (Sharma et al. 1998).

A causal relationship between exposure to biomass fuel smoke and TB is biologically plausible. The smoke could affect either risk of infection or risk of disease in infected people, or both, as has been shown to be the case with tobacco smoking (Bates et al. 2007). Without knowledge of the time of infection, however, the present study cannot distinguish between the two possibilities. Inhalation of respirable particles and chemicals found in smoke from these sources generates an inflammatory response and impairs the normal clearance of secretions on the tracheobronchial mucosal surface, and may allow TB bacteria to escape the first level of host defenses, which prevent bacilli from reaching the alveoli (Houtmeyers et al. 1999). Smoke also impairs the function of pulmonary alveolar macrophages, an important early defense mechanism against bacteria (Health Effects Institute 2002). Alveolar macrophages isolated from the lungs of smokers have reduced phagocytic ability compared with macrophages from nonsmokers and secrete a lower level of proinflammatory cytokines (Sopori 2002). Exposure to wood smoke in rabbits has been shown to negatively affect antibacterial properties of alveolar macrophages, such as their ability to phagocytize bacteria (Fick et al. 1984).

## Conclusion

Our study provides evidence that the use of biomass fuel for household heating is a risk factor for TB, but little evidence that the use of biomass as a cooking fuel is a risk factor in this population. The association is biologically plausible and consistent with the results of some other epidemiologic studies. Nonetheless, there is the possibility of a selection bias arising from differences in the sources of cases and controls. The study also strongly suggests that kerosene fuel burning, particularly for lighting, is a risk factor for TB. That kerosene lamp burning was more strongly associated with TB than kerosene stove use may be because lamps are likely to be kept burning for longer periods than are stoves, which are used only during the period of cooking, and the lamps may be kept closer to people during the evening, increasing the effective intake fraction. In addition, most of the kerosene lamps were wick lamps (21 of 22), whereas most (33 of 42) of the stoves were pressurized (pumped), which produce fewer emissions per unit fuel. Because these kerosene findings are apparently unique, more studies in different settings are needed to confirm them. Should the association with kerosene lamp use be confirmed, replacement of the kerosene lamps with solar lamps or other clean lighting systems would be a solution. Considering the strong associations of both religion and district of residence in this study, in any future case–control study examining this issue in Nepal, consideration should be given to matching on these factors.

Irrespective of the evidence for associations between indoor biomass use and TB, it is clear that such use produces substantial indoor air pollution with health-damaging chemicals and particulate matter. One, at least partially effective, remedial measure is to replace unflued stoves with chimney stoves. Such stoves, however, require continuing maintenance to maintain good indoor air quality, and because they usually just exhaust emissions to the near outdoors but not reduce them, even well- operating chimney stoves can only partly reduce total exposures (McCracken et al. 2009; Smith et al. 2009). Ideally, electric stoves or low-emission biomass stoves, such as semi-gasifier stoves, or those with cleaner burning fuels (biogas or LPG) would be used. It is more difficult to generalize about kerosene stoves and lamps, because emissions vary greatly by type of device and fuel quality, which is not uniform (Smith 1987). Pressurized kerosene stoves and lamps using good-quality fuel may have low particulate emissions if properly maintained, but inexpensive wick lamps can be dirty, particularly with low-quality fuel. Their replacement with cleaner burning devices may also be justified.

## Figures and Tables

**Table 1:** Characteristics of TB cases and controls, Pokhara, Nepal.

Characteristic	Cases (%)^a^	Controls (%)^a^	Univariate OR (95% CI)
All participants	125 (100)	250 (100)	—

Age (years)			
20–29	54 (43.2)	108 (43.2)	—
30–39	26 (20.8)	52 (20.8)	—
40–49	22 (17.6)	44 (17.6)	—
50–59	3 (2.40)	6 (2.40)	—
≥ 60	20 (16.0)	40 (16.0)	—
Mean ± SD	35 ± 13	35 ± 13	—

Residence in Kaski district			
Yes	90 (72.0)	236 (94.4)	1.00
No^b^	35 (28.0)	14 (5.60)	6.56 (3.37–12.8)

Area of residence			
Urban/periurban	87 (69.6)	212 (84.8)	1.00
Rural	38 (30.4)	38 (15.2)	2.44 (1.46–4.08)

Education			
Literate	61 (48.8)	154 (61.6)	1.00
Illiterate	64 (51.2)	96 (38.4)	1.68 (1.09–2.60)

Religion			
Hindu	89 (71.2)	236 (94.4)	1.00
Buddhist	31 (24.8)	9 (3.60)	9.13 (4.18–19.9)
Christian	4 (3.00)	5 (2.00)	2.65 (0.75–9.38)
Muslim	1 (0.01)	0 (0.00)	—

Occupation			
Government services and commerce	16 (12.8)	32 (12.8)	1.00
Farming	41 (32.8)	57 (22.8)	1.44 (0.70–2.96)
Nonagricultural labor	9 (7.20)	47 (18.8)	0.38 (0.15–0.97)
Teacher and student	11 (8.80)	15 (6.00)	1.47 (0.55–3.92)
Housewife	48 (38.4)	99 (39.6)	0.97 (0.49–1.94)

Present house construction			
* Pucca* or semi-*pucca*^c^	66 (53.0)	171 (68.0)	1.00
* Kuccha* house^d^	59 (47.0)	79 (32.0)	1.93 (1.25–3.00)

Always lived in the present house			
Yes	38 (30.4)	111 (44.4)	1.00
No	87 (69.6)	139 (55.6)	1.83 (1.16–2.88)

Crowding			
≤ 3 people per room	104 (83.2)	206 (82.4)	1.00
> 3 people per room	21 (16.8)	44 (17.6)	0.95 (0.53–1.67)

Age started cooking			
> 13 years	74 (59.2)	129 (51.6)	1.00
≤ 13 years	51 (40.8)	121 (48.4)	0.73 (0.48–1.13)

Current fuel and stove use			
Gas (GFS)	41 (32.8)	126 (50.4)	1.00
Kerosene (KFS)	19 (15.20)	23 (9.20)	2.54 (1.26–5.12)
Biomass (BFS)	65 (52.0)	101 (40.4)	1.98 (1.24–3.17)

Main heating fuel use in the house			
Electricity	1 (0.8)	3 (1.20)	—
No heating fuel	38 (30.4)	137 (54.8)	—
Combined	39 (31.2)	140 (56.0)	1.00
Wood	85 (68.0)	109 (43.6)	—
Coal	0 (0.00)	1 (0.40)	—
Kerosene	1 (0.8)	0 (0.00)	—
Combined	86 (68.8)	110 (44.0)	2.81 (1.78–4.42)

Kitchen location			
Open air kitchen and separate kitchen outside	18 (14.4)	37 (14.8)	1.00
Partitioned kitchen inside house	45 (36.0)	134 (53.6)	0.69 (0.36–1.33)
Nonpartitioned kitchen inside house	62 (49.6)	79 (31.6)	1.61 (0.84–3.10)

Windows in the kitchen			
Yes	117 (95.1)	231 (92.8)	1.00
No	6 (4.90)	18 (7.20)	0.66 (0.25–1.70)
Missing	2	1	

Overall ventilation in the kitchen			
Fully ventilated	59 (47.2)	161 (64.4)	1.00
Unventilated	66 (52.8)	89 (35.6)	2.02 (1.31–3.13)

Source of light in the house			
Electricity	107 (85.6)	246 (98.4)	1.00
Kerosene lamp	18 (14.4)	4 (1.6)	10.35 (3.42–31.3)

Smoking status			
Never smoked	83 (66.4)	200 (80.0)	1.00
Ever smoked	42 (33.6)	50 (20.0)	2.02 (1.25–3.28)

Pack-years of smoking			
0	84 (66.2)	200 (80.0)	1.00
≤ 8	16 (12.8)	31 (12.4)	1.23 (0.64–2.37)
> 8	25 (20.0)	19 (7.60)	3.13 (1.64–5.99)

Smokers in the family			
None	58 (46.4)	165 (66.0)	1.00
One	48 (38.4)	72 (28.8)	1.90 (1.18–3.04)
Two or more	19 (15.2)	13 (5.20)	4.16 (1.93–8.95)

Burn mosquito coils indoors			
No	76 (60.8)	136 (54.8)	1.00
Yes	49 (39.2)	113 (45.2)	0.78 (0.50–1.21)
Missing	0	1	

Burn incense indoors			
No	28 (22.4)	46 (18.4)	1.00
Yes	97 (77.6)	204 (81.6)	0.78 (0.46–1.32)

Alcohol consumption			
No	106 (85.5)	238 (95.6)	1.00
Yes	18 (14.5)	11 (4.40)	3.67 (1.68–8.05)
Missing	1	1	

Taking vitamin supplements			
No	120 (97.56)	214 (85.94)	1.00
Yes	3 (2.44)	35 (14.06)	0.15 (0.05–0.51)
Missing	2	1	

Household member had TB			
No	77 (61.6)	227 (90.8)	1.00
Yes	48 (38.4)	23 (9.20)	6.15 (3.51–10.8)

Annual income (Nepalese rupees)			
≤ 25,000	26 (23.9)	72 (30.3)	1.00
25,000–50,000	58 (53.2)	90 (37.8)	1.78 (1.02–3.13)
> 50,000 to ≤ 100,000	16 (14.7)	51 (21.4)	0.87 (0.42–1.79)
> 100,000	9 (8.20)	25 (10.5)	0.99 (0.41–2.42)
Missing	16	12	

Land ownership			
No	32 (25.6)	83 (33.3)	1.00
Yes	93 (74.4)	166 (66.7)	1.47 (0.91–2.38)
Missing	—	1	

Personal transportation			
Yes	15 (12.0)	47 (18.8)	1.00
No	110 (88.0)	203 (81.2)	1.70 (0.91–3.17)

a No missing data, except as indicated.

b Tanahu, Syangja, Baglung, Parbat, Myagdi, and Lamjung districts.

c *Pucca* house made with brick and cement; semi-*pucca* house made with brick and mud.

d *Kuccha* house made with bamboo and mud (with thatched roof).

**Table 2:** Multivariate logistic regression model for fuel use in relation to TB in women in Pokhara, Nepal (log likelihood = − 118.73, *R* ^2^ = 0.44).

Variable	OR (95% CI)^a^
Fuel stove	
GFS	1.00
BFS	1.21 (0.48–3.05)
KFS	3.36 (1.01–11.22)
Heating fuel	
No heating fuel use or electricity	1.00
Biomass, coal, or kerosene	3.45 (1.44–8.27)
Main light source in the house	
Electricity	1.00
Kerosene lamp	9.43 (1.45–61.32)

a Adjusted for age, religion, income, residence locality, residence district, literacy, type of present house construction, always lived in the present house, pack-years of smoking, number of family members who smoked indoors, alcohol consumption, taking vitamin supplements, family history of TB, and ventilation in the kitchen.

**Table 3:** Exposure–response relationships based on duration of cooking with BFS and KFS.

			OR (95% CI)	
Exposure to fuel stove	Cases (%)	Controls (%)	Adjusted^a^	Unadjusted
Exposure to BFS (years)				
0	26 (20.8)	43 (17.2)	1.00	1.00
> 0 to ≤ 5	20 (16.0)	28 (11.2)	1.17 (0.32–4.32)	1.18 (0.55–2.52)
> 5 to ≤ 10	18 (14.4)	51 (20.4)	0.64 (0.18–2.20)	0.58 (0.28–1.22)
> 10	61 (48.8)	128 (51.2)	0.47 (0.11–2.02)	0.79 (0.44–1.40)

Exposure to KFS (years)				
0	86 (68.8)	209 (83.6)	1.00	1.00
> 0 to ≤ 5	12 (9.6)	14 (5.60)	4.96 (1.44–17.1)	2.09 (0.93–4.73)
> 5	27 (21.6)	27 (10.8)	4.60 (1.34–15.7)	2.54 (1.39–4.64)

a Adjusted for duration of use of BFS and KFS, GFS, biomass heating fuel, ventilation, use of kerosene lamp, pack-years of smoking, number of family members smoking indoors, religion, residence district, locality, literacy, present house construction, always lived in the present house, alcohol consumption, family members had TB in the past, taking vitamin supplements, income, and age.
